# A Revised Concept for Ocular Surface Imprinting: Easy-to-Use Device for Morphological and Biomolecular-Based Differential Diagnosis

**DOI:** 10.3390/diagnostics15202660

**Published:** 2025-10-21

**Authors:** Bijorn Omar Balzamino, Ilaria Ghezzi, Roberto Sgrulletta, Rossella Anna Maria Colabelli Gisoldi, Augusto Pocobelli, Antonio Di Zazzo, Loredana Zollo, Alessandra Micera

**Affiliations:** 1 Research Laboratories in Ophthalmology, IRCCS–Fondazione Bietti, 00184 Rome, Italy; bijorn.balzamino@fondazionebietti.it; 2 Laboratory of Biomedical Robotics & Biomicrosystems, University Campus Biomedico, 00128 Rome, Italy; i.ghezzi@unicampus.it (I.G.); l.zollo@unicampus.it (L.Z.); 3 Department of Ophthalmology, University Campus Biomedico, 00128 Rome, Italy; r.sgrulletta@unicampus.it (R.S.); a.dizazzo@unicampus.it (A.D.Z.); 4 Eye Bank, San Giovanni-Addolorata-Britannico Hospital, 00184 Rome, Italy; ramcolabelligisoldi@hsangiovanni.roma.it (R.A.M.C.G.); apocobelli@hsangiovanni.roma.it (A.P.)

**Keywords:** point-of-care device, clinical practice, epithelial imprints, sampling device, ocular surface, goblet cells

## Abstract

**Background/objectives:** The continuous necessity to support biostrumental data with biolomecular data collected using non-invasive tools is influencing the world of ocular surface devices. The ocular imprint still represents a non-invasive and safety technique for collecting corneal and conjunctival epithelia in an easy way, as performed in human and veterinary clinics. Although used in clinical practice since 1977, operators might benefit from improvements in these techniques, especially in terms of handling and management. **Methods:** Herein, by reporting the design and characteristics of a patent of ocular surface sampling (the SurfAL pen and periocular-assisted SurfAL pen; PCT WO2016IB51474 20160316), we performed a validation and analysis of its value compared to gold standards. The level-headedness and advantages of this device were verified in 15 sclerocorneal specimens (sampling advantages) and tested in 25 volunteers (handling and operator efficiency, as well as frequency of discomfort in volunteers). Morphological as well as biomolecular analyses were used to compare SurfAL devices with conventional ones. **Results:** The easy management of SurfAL pens and the good detection of epithelial/goblet cells were confirmed. The SurfAL pen was found to be smart and suitable for routine analysis, as confirmed by quick and reproducible onsite sampling. Periocular-assisted SurfAL pen was comparable in terms of sampling quality but less comparable in terms of subject confidence due to its geometry. **Conclusions:** This study suggests that the SurfAL pen and periocular-assisted SurfAL pen might represent an additional and hands-on way of sampling ocular surface cells and improve the diagnostic route in ophthalmology.

## 1. Introduction

The role of the ocular surface (OS)—corneal epithelium, tear film, nerves—in the homeostasis of the entire eye globe is undeniable. OS is daily exposed to the environment and the presence of tidy, regulated, and efficient action performed by mechanical, physical, and biochemical strategies, main aspects to guarantee local homeostasis and function [1,2]. Differential diagnosis of OS disease (dry-eye syndrome vs. dry-eye disease (DES vs. DED), bacterial to fungal infection (acanthamoeba vs. chlamydia), which can share common targets and signature of inflammation, and appears complex to diagnose with the “lone” biostrumental support [3,4,5]. OS is also influenced by epigenetic factors and lifestyle habits, making it difficult to discriminate in the case of subclinical inflammatory states (urban city) [6,7,8]. Corroborating data suggests that aging and the type of work (video terminal operators, librarians, clerks) may influence ocular surface inflammation [3,9].

To date, OS diseases can be viewed as a complex group of disorders requiring an emergency approach sustained by quick and correct diagnosis, especially in the presence of mandatory cases for differential screening or cellular characterization [10]. A routine diagnosis of OS involves using laboratory tests and instruments that collect information on tears and OS, combined with biomolecular and morphological information [11,12,13]. The concept of “Impression Cytology” or just “imprinting” encompass a series of techniques devoted to the collection of few epithelial layers and inflammatory cells (if enough superficial) by a peel-off approach, and devoted to the differential diagnosis throughout the evaluation of few cell parameters, from morphological (distribution/density of goblet cells) to biomolecular ones, including structural and few immune resident/infiltrating immune cells whenever pathologically present [14,15]. Due to the necessity to have quick and efficient differential diagnosis for prompt therapy, a continuous need for less invasive and easy-to-use in-office methods is warranted. Some routine procedures are performed by cytological tests carried out at the end of ophthalmological examination [14,15]. Scraping/brushing of the OS is possible in case of injured and/or inflamed areas of conjunctiva and/or cornea, such as in case of infection-based keratitis, but these tools do not represent an optimal way of sampling in case of intact OS [11,13]. The concept behind the collection of a few epithelial cells by means of a local “imprint” occurred as early as 1954 for the diagnosis of a squamous neoplasm [14] and was confirmed in 1977 by using a cellulose membrane to quantify the presence of goblet cells in patients with dry eye syndrome [15]. The procedure is frequently performed, although some limitations of the current methods are known. Sometimes the sampling is not suitable for the analysis, meaning that the peel-off could not be accurate (low or too high), as a result of inadequate membrane pressing at the ocular surface [15]. If quickly and appropriately performed, the procedure guarantees a good preservation of conjunctival and/or corneal cell morphology and allows unlimited use of cytodiagnosis techniques, from the simplest (morphology) to the complex (immunocytochemistry, immunofluorescence, flow-cytometry, real-time PCR, and protein array) ones [4,16,17,18].

Today, there are two main patents for OS imprinting, both smart and non-invasive, devoted to microscopical and biomolecular analysis, based on the new nitrocellulose Biopore membrane (0.22 µm; MilliCell Sterilized Culture Plate Insert, Millipore Corp., Bedford, MA, USA) [19,20]. The usage of nitrocellulose appears to be the main one, consistent with a high number of ocular surface applications (dry eyes, vitamin deficiencies, pemphigus, ocular scarring, limb deficits, viral/bacterial infections, allergic disorders, and conjunctival melanomas) [16,21,22].

Herein, we describe the concept of SurfAL and periocular-assisted SurfAL (shortened as periocular or combo SurfAL) and produce morphological as well as biomolecular data on its validation.

## 2. Materials and Methods

### 2.1. Patent Device Description

The device granted the PCT (WO2016147122A1) and recently received the US 2018/0070929 A1, 15 March 2018 [19], “Device for sampling of the eye surface by imprinting” (Inventors: Micera A, Zollo L, Ghezzi I, Balzamino BO, Sgrulletta R. Applicants: IRCCS-Fondazione Bietti and Campus BioMedico, Rome, Italy).

**Figure 1:**
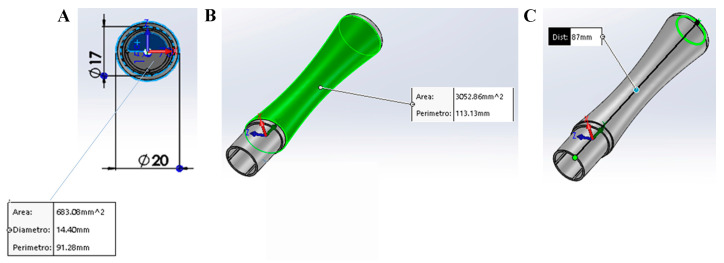
Description of SurfAL pen. (**A**) Front view of the hollow for the insertion of the MilliCell membrane support or the single membrane. Dimensions are summarized in the box. (**B**,**C**) Pick-up pen: stick and dimensions. As shown in A, the SurfAL pen has a full cylindrical shape (17 mm outer diameter). The 14.5 mm inner diameter can hold the nitrocellulose membrane (Millicell, Millipore). The total length is approximately 87 mm, and the weight is about 10 g, allowing a perfect grip for the operator’s hand. Mounting geometry has been designed to be easy to handle for operators. Depending on packaging, the device can be disposable or refillable with the commercial membrane device.

The device includes two main parts: device A, a pen equipped with a membrane cap (referred to as the SurfAL pen), and device B, a periocular-assisted SurfAL pen device for a more accurate sampling (shortened as periocular device). SurfAL pen is designed to be used individually (clinical sampling) or coupled to the Periocular device that allows assisted sampling of defined ocular surface (upper, lower, temporal, and nasal) areas, merely at surgery (combo SurfAL). The original design of both single and coupled parts was developed using Version 23—SolidWorks 2015—Released 9 September 2014 (Dassault Systèmes, Concord, MA, USA), eliminating possible errors and reducing time/materials prior to building. The prototypes were built using a 3D Objet500 Connex printer (Stratasys Ltd., Minneapolis, MN, USA) using ABS (acrylonitrile butadiene styrene) material (Ultimaker B.V., Geldermalsen, the Netherlands) and used for validation tests. These fast-manufacturing techniques included a 3D printer that allowed photo-polymer jet depositing resins with layers of 16–30 µm with a print precision of 0.1–0.3 mm. The production of the prototype was made of “True White”, a 30 µm slicing material that offers hardness, dimensional stability, and excellent visibility to details. Finally, this material is suitable for medical strategies. The characteristics of SurfAL pen are shown in Figure 1A–C.

**Figure 2:**
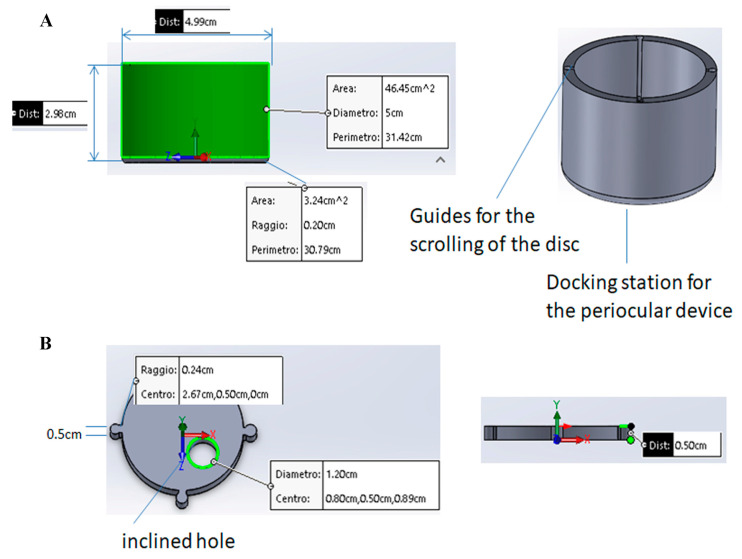
(**A**) The periocular-assisted SurfAL pen device (combo device) has a cylindrical geometry with a disk moving on four linear rails containing specific springs. The device controls the imprinting force on the ocular surface. The round shape (50 mm outside and 40 mm inside) provides a better adherence to the ocular orbit and a complete wrapping of the eye orbit in a comfortable condition for the patient. The eyelid motion is partially blocked, and the device does not cause any sense of discomfort or constriction to the patient. The height is approximately 30 mm, and the weight is about 50 g, excluding the inner springs. (**B**) Upper view of the disk with a hole for pick-up stick insertion. Related measurements are reported. The mobile disk has one hole of 17 mm to allow the insertion of the SurfAL pen, as highlighted in green, allowing sampling at four different quadrants on the eye surface. To compensate for the curvature of the eye, avoiding incorrect inclinations of the SurfAL pen, the hole is inclined at an angle of 10° to the center of the disk.

While the SurfAL pen was designed for a quick and easy sampling, the Periocular device was conceived to allow a targeted and reproducible sampling of defined areas, reducing both hand tremors and inexpert actions by the operator (Figure 2A,B).

### 2.2. Study Population: Ethical Approach and Sampling Procedures

**Figure 3:**
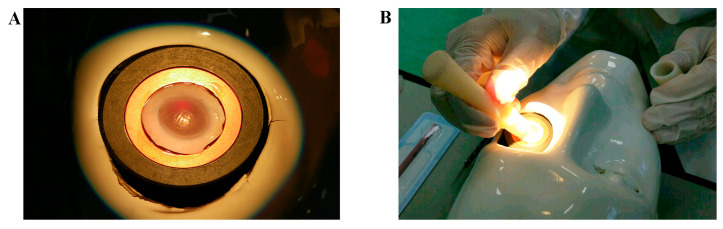
Sampling procedures. (**A**) A particular corneoscleral rim in the holder, as backlit by optic fibers. (**B**) a time-lapse of the sampling procedure. Both time, pressure and inclination were reproduced as in Clinique.

The study design was approved by both research institutions (Campus Biomedico of Rome and Fondazione Bietti) and was conducted in accordance with the standards stated in the Declaration of Helsinki and in line with national and international guidelines. Prot. 15/18TS 11.06.2018, Date: 11 June 2018, and Prot. 40/13 PAR 25.06.2013 ComEt CBM, Date: 25 June 2013. For ex vivo experiments, 15 sclerocorneal specimens (10F/5M; mean age 69.5, ranging 65–74 years), a surplus from experimental teaching, were obtained from the Eye Bank (Ospedale Addolorata San Giovanni, Rome, Italy). All tests were performed by an ophthalmologist in an experimental room (wet lab) equipped with a light microscope, digital color core units (OPMI 1-FC Zeiss; Carl Zeiss, Oberkochen, Germany), and a video camera (ExwaveHAD; Sony, Minato, Tokyo, Japan) placed on small benchtops. The particular sclerocorneal rim holder is depicted in Figure 3A, while a moment of the SurfAL pen sampling procedure over a corneoscleral rim placed on the mannequin face is illustrated in Figure 3B.

The in vivo tests, 20 volunteers (3 biologists; 2 engineers, 3 ophthalmologists, 2 students, and 10 volunteers; 8M/12F, age range 25 to 61 years old) joined the study and provided a fully written informed consent.

Imprints were collected by i. gold-standard (Millipore membranes) [22], ii. SurfAL pen and iii. periocular-assisted SurfAL pen system. Samplings were performed with or without local anesthesia (4 mg/mL (0.4%) Novesin; Thea, Novartis Farma SpA, Basel, Switzerland), strictly reliant on specific volunteers’ requests. Imprints were performed only at the conjunctiva. The procedure included gently applying the membrane onto the conjunctiva for 5 s to provide a right peel-off. Prolonged pressure was accurately avoided, as associated with tearing and therefore a reduced quality of sampling.

### 2.3. Validation Analysis: Handling Performance, Morphological, and Biomolecular Tests

#### 2.3.1. Handling and General Performance

A questionnaire was prepared to assess various properties of the device, including usability, flexibility, user preferences, and user acceptance. It provided a way to obtain feedback on the SurfAL device’s performance, ease of use, and overall satisfaction.

Membranes were quickly fixed (Citofix; Bioptica, Milan, Italy), removed from the plastic support, and placed on a glass slide for specific staining (basal histology or Immunofluorescence) or extraction (mirVana PARIS; Thermo Fisher Scientific, Waltham, MA, USA). A scalpel (DIEMME Dispositivi Medici S.r.l., Milan, Italy) was used to separate membranes from plastic supports.

#### 2.3.2. Microscopy Analysis

Prefixed cytologies were processed for PAS (Periodic Acid-Schiff) staining followed by hematoxylin counterstain, with minor modifications developed for staining cell monolayers on nitrocellulose membranes (04-130802; Bioptica, Milan, Italy). Prefixed cytologies were briefly equilibrated in phosphate-buffered saline (PBS) (10 mM phosphate buffer and 137 mM NaCl; pH 7.5), blocked/permeabilized with 0.1% bovine serum albumin (BSA)/0.3% Triton X100 in PBS before quick quenching (10 mM NH4Cl) and probing with the antibodies listed in Table 1A. The specific binding was detected using Cy2/Cy3-conjugated species-specific secondary antibodies (1/500–1/700; Jackson ImmunoResearch Labs., Europe Ltd., Suffolk, UK). Nuclei were counterstained with Propidium Iodide (10 mM; Invitrogen-Molecular Probes, Eugene, OR, USA). Internal controls were carried out (control irrelevant IgGs; Vector Laboratories, Inc., Burlingame, CA, USA) and used for noise reduction (channel-series setup; Nikon). Acquisitions were carried out using the direct epifluorescent microscope (TE2000U) equipped with light/fluorescent route and NIS 4.0 software (Nikon, Tokyo, Japan). For integrated optical density (IntDen), the mean ± SD data (optic field) were calculated, grouped, and subjected to statistical analysis. The 8-bit TIFF saved digital images (512 × 512 or 1024 × 1024 dpi; *n* = 5 areas/membrane; ×40/dry 0.75 DIC M/N2) were subjected to single fluorescent acquisition, and MFI values were produced by the freely available ImageJ v1.43 software (NIH). Digital images and graph plots were assembled by using Adobe Photoshop 2024 (Adobe Systems Inc., San Jose, CA, USA). Antibodies used for immunofluorescence are listed in Table 1A.

#### 2.3.3. Biomolecular Analysis: All-in-One RNA and Protein Extraction

Membranes were immediately processed with a mirVana PARIS kit (ThermoFischer, Waltham, MA, USA) to collect both RNA and proteins from each sample. To increase the efficacy of cell release, a preincubation with 0.5% Triton in PBS was performed for 1 h under shaking.

PCR analysis: RNA was normalized through the spectrophotometer (ND1000; λ260/280 > 1.8; NanoDrop Technologies, Wilmington, DE, USA) and screened with agarose gel size-fractionation analysis. total RNA (3 µg) was used for cDNA synthesis, using Moloney Murine Leukemia Virus (MMLV) reverse transcriptase (IMPROM; Promega, Milan, Italy). For amplification, SYBR Green PCR Master Mix (Applied Biosystems, Foster City, CA, USA) was used, and 40 cycles with specific primers were run on a 48-well Eco Illumina platform (Illumina, Inc., San Diego, CA, USA) [23]. For molecular analyses, all comparisons were carried out considering the Ct values produced at the end of amplification. Ct values are inversely proportional to transcript expressions. Primers and PCR amplification protocol are reported in Table 1B. Negative (no template) and positive controls were produced for each plate run. [24].

Western blot analysis: Native proteins were further diluted in modified RIPA (50 mM Tris-HCl, 150 mM NaCl, 1 mM EDTA, 0.1% Nonidet P-40, 1 mM NaF, and 1 mM PMSF; pH 7.5), briefly sonicated (VibraCell; Sonics, Newtown, CT, USA), clarified by centrifugation (13,000 rpm/7 min) and analyzed (3 µL) with the A280 program (Nanodrop; Celbio, Milan, Italy). A total of 30 µg of normalized samples were boiled in loading buffer (100 mM Tris–HCl buffer containing 0.2 M DTT, 0.5% SDS, 20% glycerol and 0.1% bromophenol blue; pH 6.8), separated under reducing conditions on 4–20% mini-SDS polyacrylamide gels (SDS-PAGE; 130 V/frontline; Miniprotean3 apparatus; Biorad, Hercules, CA, USA) and transferred to Hybond membranes (GE Healthcare, Buckinghamshire, UK) under semidry conditions (13 V/35 min; semidry transblot system; Biorad). Prestained broad weight marker was run in parallel (10–250 kDa prestained marker; Biorad). Bands were visualized by Ponceau S. Membranes were blocked in 5% BSA in TBS-TW (10 mM Tris—100 mM NaCl containing 0.1% Tween-20; pH 7.5) for 60 min at room temperature and subsequently probed with human anti-CK19 (1:100) and human anti-actin (1:1000) antibodies and then incubated with horseradish peroxidase-conjugated secondary antibodies (1:10,000, 45 min, under gentle shaking). Specific binding was developed with ECL chemiluminescent substrate (Millipore, Burlington, MA, USA). Integrated Density (IntDen) was calculated by ImageJ Software. Optical density acquisition of Actin bands was used as an additional normalizing factor, and IntDen values were used for comparisons. Data are expressed as a percentage of expression in normalized controls. Antibodies used for Western blot are listed in Table 1A.

### 2.4. Statistical Analysis

Appropriate statistical comparisons were carried out with Statview (Abacus Corporation, California, CA, USA) or Prism 10.2 (GraphPad Software, San Diego, CA, USA). For questionnaire evaluation, the chi-square (χ2) test was carried out to understand the quality of the device and the combo device. For microscopy evaluations, two unmasked operators were used to count positive cells, and optic fields (3 diagonal areas/imprint) were selected to analyze each membrane by using a 10 × 10 ocular grid (×20 magnification). For biomolecular evaluations, ANOVA analysis was used to compare protein expressions between subgroups, while REST–ANOVA coupled analysis was carried out for identifying significant changes in real-time PCR experiments.

Data are shown as mean ± SD or median ± SD, depending on the graphical representation. The preliminary evaluation of the normal distribution of data was performed by the Kolmogorov–Smirnov (KS) and the Shapiro–Wilk (SW) tests. Normality check suggested the use of ANOVA followed by the Tukey–Kramer post hoc analysis. Significant levels were: * *p*-value ≤ 0.05; ** *p* < 0.01; *** *p* < 0.001; and **** *p* < 0.0001. For molecular analyses, the correction for total biomarkers was also applied, considering 10 biomarkers (*p* = 0.005).

## 3. Results

**Figure 4:**
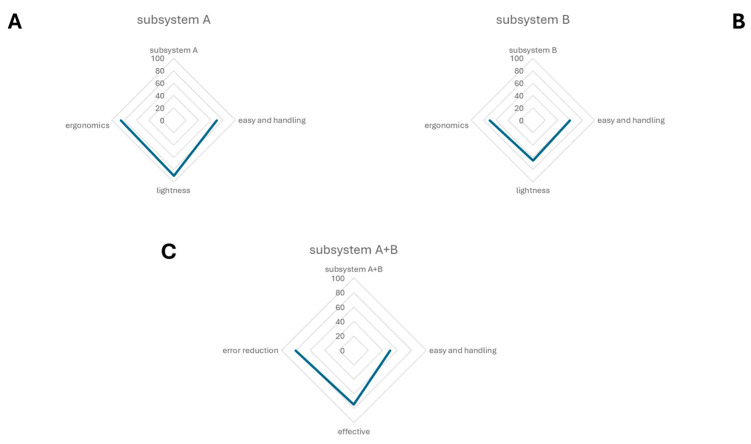
A radar chart showing the results of the analysis of questionnaires. This spider chart represents the allocated qualities of the two separated subsystems and the use of the combo device. The radar chart shows the differences in performance metrics of subsystem A (**A**), subsystem B (**B**) and the combo subsystem A + B (**C**).

As introduced above, a questionnaire was prepared to test satisfaction with the usage of this device. Principal components analysis of the specialists’ assessments revealed three main factors of satisfaction: the easy handling of the device; the possibility to use and calibrate the force to collect corneal imprints and the reaction of patients to the sampling; and the perceived length of sampling (Figure 4). The SurfAL device was analyzed for its physical and mechanical characteristics as subsystem A (A), subsystem B (B), and the combo-assisted subsystem A + B (C). Volunteers (sanitary service users) expressed their own opinion regarding the properties of the device and perceptions, having also tested the EYEPRIM™ device and the Millicell insert. The satisfaction scale was divided into insufficient, sufficient, good, and excellent. The analysis of questionnaires developed specifically for the device analysis showed that—85% of operators surveyed considered subsystem A lightweight, ergonomic, and easy-to-use and easy-to-handle. Users had no comments regarding shaping or material features, but expressed interest in using it. Only sixty percent (60%) of users showed scarce interest in subsystem B, considering it bulky or its geometry unsuitable for their use. However, users recognized the potential improvements in the sampling procedure, and greater efficiency and simplicity. On the whole, approximately seventy percent (70%) of users were satisfied with the proposed device and rated it easy to use, giving it an overall rating of good/excellent. Volunteers’ comments were not devoted to reducing the qualities and usefulness of EYEPRIM™ but highlighting the potential aspects of SurfAL. Finally, this study highlighted that the SurfAL subsystem A device by itself is more ergonomic, lightweight, and flexible than the EYEPRIM™ device. The device was found to be easy to use because, not being a “syringe,” the operator does not need to apply pressure that could destabilize the hand upon impact with the ocular surface, especially in the case of inexperienced users. This aspect makes the device useful for those inexperienced in the field. Furthermore, the device has a significant environmental impact because, if desired, only the membrane is replaceable. The device does not come into direct contact with the patient, as caps (membrane supports) can be provided in a single, mono-wrapped, sterilized form, and the user can bring the rechargeable disposable for in-office procedures. On the other hand, caps can be more flexible when sending the device to the laboratory for molecular analysis. Regarding the point-of-care finality, SurfAL does not join a buffer system; however, a reaction box can be provided on demand to allow an on-site evaluation of the morphological features of the imprinted monolayer.

### 3.1. Device Performance in Clinical Practice

**Figure 5:**
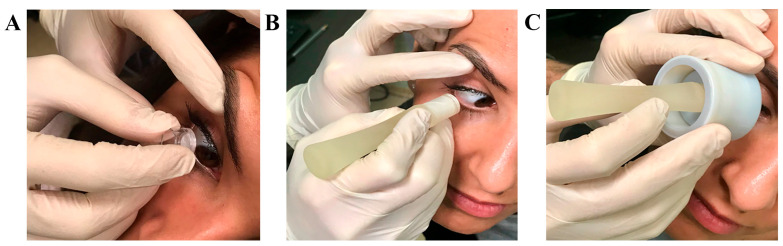
Sampling procedures. From left to right are the following procedures(**A**,**C**): (**A**) Millicell support; (**B**) SurfAL pen; (**C**) periocular-assisted SurfAL device (combo SurfAL). The volunteer adhered to illustrative purposes.

The different ways of sampling are shown in Figure 5A–C: a conventional Biopore membrane device (8 mm diameter; 0.4 µm Biopore filter, MilliCell, Figure 4A); the nitrocellulose membrane on SurfAL device (Pall Corporation, Port Washington, NY, USA; Figure 4B); and the periocular-assisted SurfAL device shown in Figure 4C.

PAS staining showed that all imprints were of good cell density and consisted of epithelial cells (faded pink) with different grades of mucilage staining (magenta/violet). When comparing to conventional sampling (Figure 5A), SurfAL pen collection and combo SurfAL produced good quality staining with well-defined epithelial cell layers and well-distributed cells (Figure 5B,C).

### 3.2. Device Performance in Laboratory Routine: Microscopy and Real-Time RT-PCR

**Figure 6:**
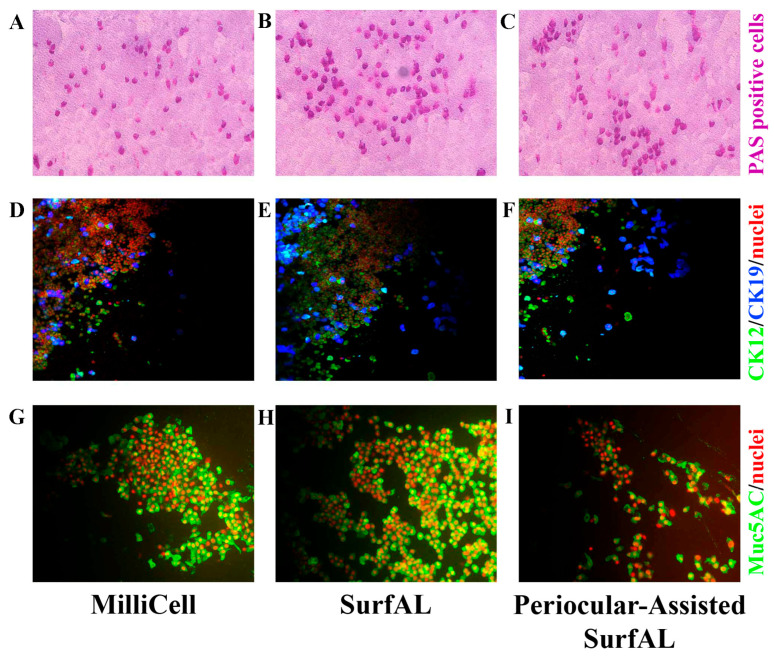
Microscopy validation. Imprints from the three different methods of sampling were stained for basal cytology (PAS staining) or immunofluorescent analysis of mucins (muc5AC) and cytokeratins (CK12; CK19). PAS-positive magenta-stained goblet cells in the upper panels (**A**–**C**); CK12/19-immunoreactive (**D**–**F**) and Muc5AC-positive cells (**F**,**G**) are shown, respectively, in the middle and lower panels. The number of PAS-positive or mucin-immunoreactive cells represents an index of the healthiness of the ocular surface and is therefore used for diagnostic purposes. Rows included as follows: (**A**,**D**,**G**), MilliCell; (**B**,**E**,**H**), SurfAL pen; (**C**,**F**,**I**), periocular-assisted SurfAL pen device.

Test repeatability for cytological validation. As compared to Millicell (26.89 ± 1.92 goblet cells; *n* = 32 optic fields; Figure 6A), the differences in the number of PAS positive cells (goblet cells) between SurfAL (35.82 ± 1.73 goblet cells; *n* = 32 optic fields; Figure 6B) and periocular-assisted SurfAL (28.32 ± 1.99 goblet cells; *n* = 36 optic fields; Figure 6C) devices were not significant (*p* > 0.05, ANOVA analysis; Figure 6B,C). A trend to an increase in the number of positive cells was observed between the SurfAL and periocular-assisted one, as well as the Millicell devices. Interesting, SurfAL pen group was the only one to have a 95% confidence interval above zero (13.15%), resulting in high reproducibility. Regarding the periocular device, some empty areas were found, and the epithelium did not appear smooth, most probably due to an imperfect adaptation of the device to the orbital cavity.

**Figure 7:**
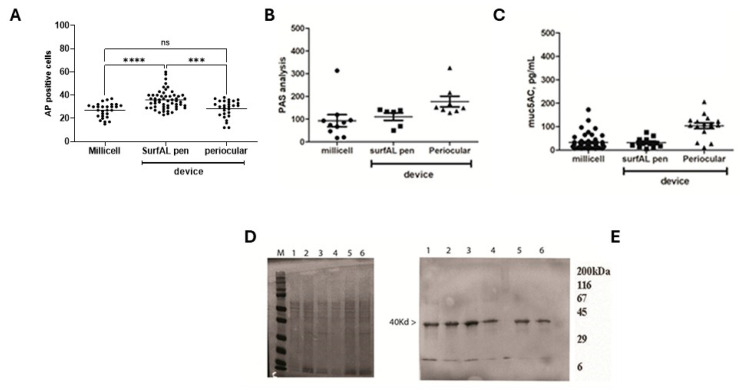
Microscopic vs. biochemical validations on imprints from the three different methods of sampling. (**A**) Cell counting of Acidic Phosphatases immunolabeled cells (**A**) and IntDen analysis (MFI) for PAS (**B**) and muc5AC (**C**) positive cells per optic field (×20), respectively, in membranes from Millicell, SurfAL pen, and periocular device. (**D**) Respectively 4–20% SDS-PAGE and (**E**) cytokeratin (CK) 19 probed immunoblot from imprints: Millicell (1,2), SurfAL pen (3,4), and periocular (5,6) devices. MFI, Mean Fluorescent Intensity; M, sizer; L, ladder. Significant changes are pointed by asterisks (ns not significant; *** *p* < 0.005; **** *p* < 0.001 REST–ANOVA–Tukey–Kramer post hoc).

Test repeatability for biochemical validation. For this task, Acid phosphatase (AP) and muc5AC as markers of ocular epithelia and tear film functionality. Immunoreactivity for CK12/19 and muc5AC showed the possibility to label structural and functional targets, as shown in Figure 6D–F. The immunofluorescent analysis specific for immunolabels CK12/19 and muc5AC was quantified, showing a comparative expression of cytokeratins (CK12; CK19) and muc5AC between devices. The quantitative analysis of both AP-positive cells (Figure 7A), PAS (Figure 7B), and muc5AC (Figure 7C) IntDen analysis is shown from specific acquisitions. Both SDS-PAGE electrophoresis and immunoblot specifically for cytokeratin CK19, a major cytokeratin for conjunctival epithelia, are reported in Figure 7D and Figure 7E, respectively.

**Figure 8:**
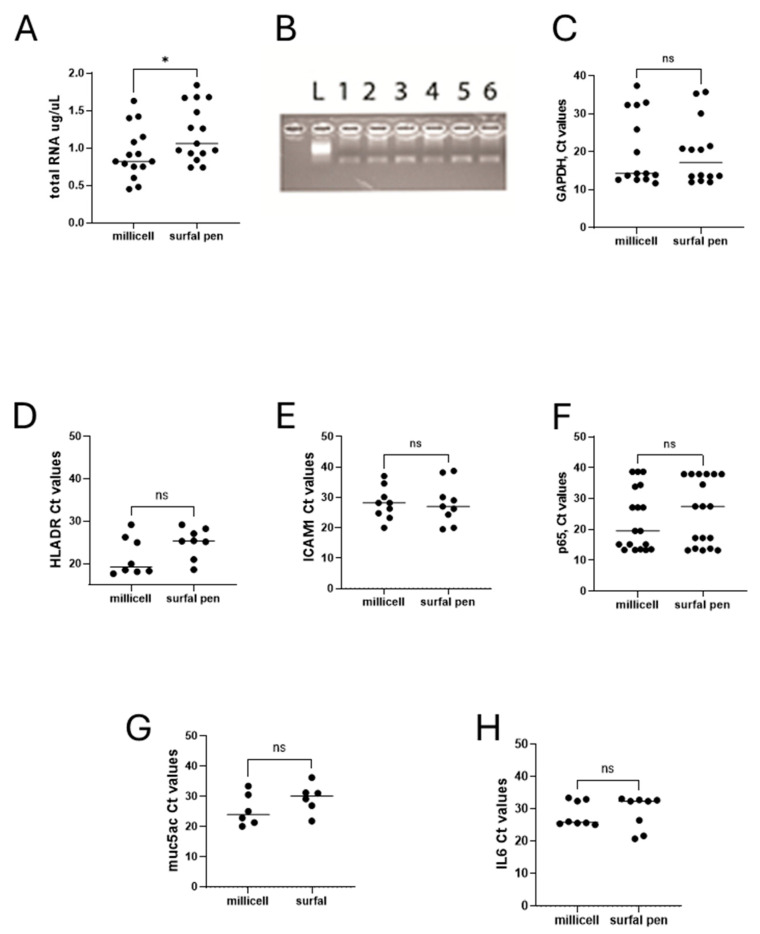
Molecular validations. Total RNA extraction and transcript amplification were carried out on imprints from Millicell and SurfAL devices. The panel shows the following: (**A**) total RNA expression in comparison between Millicell and SurfAL; (**B**) representative amplicons (muc5ACmRNA) in membranes from all devices run in duplicate (from left to right: size marker (L), Millicell (1,2), SurfAL pen (3,4) and periocular (5,6) devices); and (**C**) scatter-plots showing the Ct values for GAPDH (**C**), HLADR (**D**), ICAM1 (**E**), p65NFkB (**G**), muc5AC (**F**), and IL6 (**H**), all Millicell vs. SurfAL pen. Note that the Cts values were used for comparison. Cts and transcript expression values are inversely related. (ns not significant and * *p* < 0.05 REST–ANOVA–Tukey–Kramer post hoc).

Test repeatability for molecular validation. For the comparison of different imprints from Millicell and SurfAL, a total RNA extraction protocol was applied according to the methodology as described above. Regarding total RNA extraction efficiency, Millicell and SurfAL pen devices showed a slight but significant difference in total RNA recovery (*p* < 0.05), as quantified by a microvolume nanodrop spectrophotometer. Although significant, this difference in RNA recovery was in an average of 0.9 µg/µL total mRNA (ranging from 0.43 to 1.61 µg/µL), as detected in 21 µL final eluate (ddw). The variability in RNA recovery observed in the different samples (Figure 8A) did not influence the PCR amplification (Figure 8B) and was retained after housekeeping amplification (Figure 8C). A possible explanation for the major stability of imprinting by SurfAL. The low amounts might account for excessive tearing or dryness of the ocular surface during sampling, in the absence of a “tidy” stabilization of the handmade sampling. No significant differences were observed between Millicell and SurfAL specimens in terms of RNA purity (*p* > 0.05).

Since some targets are currently used as representative indicators of healthiness and functional ocular surface and have been repeatedly proposed for diagnostic purposes, together with the cytological markers, we compared the transcript expression of few ocular surface and tear film stability biomarkers of diagnosis and monitoring: HLADR-ICAM1-p65, as indicators of immune cell interaction and inflammation; muc5AC as markers of ocular epithelia and tear film stability, and IL6 as marker of inflammatory signaling and immune driven response. The transcript expression related to these indicators of healthiness and functionality is shown in Figure 8D–H. The comparison of Ct values from the amplification carried out on Millicell and SurfAL pen showed the absence of amplification for all targets investigated, most probably due to the fact that both devices are made of the same nitrocellulose matrix.

## 4. Discussion

**Figure 9:**
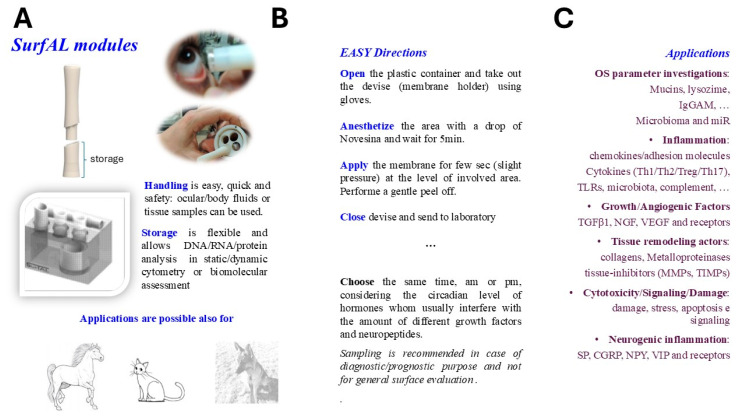
Schematic representation of SurfAL modules and applications. The SurfAL device was designed and developed to assist clinical human and veterinary practice with different diagnostic purposes. (**A**) SurfAL is a flexible platform to be used for identifying biomarkers representative of different stages of discomfort or disease whenever validated in other studies. The periocular-assisted SurfAL device is shown and allows a more flexible, easy, and precise monolayer sampling for human and veterinary practices. SurfAL loads appropriate membranes (nitrocellulose) as Millicell does. (**B**) The major points in the procedure of sampling are summarized, highlighting the easy procedure and flexibility of this point-of-care. In private clinical centers as well as in clinical and surgical departments, the point-of-care device might provide quick sampling and delivery to the laboratory for analysis or just an on-site analysis. (**C**) A list of potential targets suitable for this device, clustered for functional and morphological purposes, is shown. Biomarkers include proteins and peptides, DNA, RNA, and microRNA, as well as infections and microbiome targets, cortisol and neuropeptides, hormones, stress-related proteins, and Reactive Oxygen Species (ROS). Targets can be detected at both biomolecular and morphological levels. A simultaneous detection of biomarkers can be suitable (RNA/DNA/protein), and the morphological analyses represent an additional point for point-of-care applications.

Herein, the study shows the features of the device and the validation data supporting the detection of specific alterations of the ocular surface to support the differential diagnosis in ophthalmology. Overall, device characteristics and application are schematized in Figure 9.

Precision medicine and personalized therapy need the combination of both imaging and biomarker examination [25,26]. Smart and hands-on devices, not necessarily of a high technological level, simplify the way of sampling. Merely in ophthalmology, there is a strong need for portable devices to provide onsite diagnosis and/or monitoring and prognosis with a significant reduction in waiting time due to hospital procedures.

Clinical implications of the quick detection of the OS alterations are critical for good therapy [27,28]. This aspect is particularly important for those cases associated with infections [18]. Effective therapy should involve a combination of medications targeting both the active (trophozoite) and dormant (cyst) forms of the parasite in a quick and specific fashion [29]. An early detection might also be crucial for Chlamydia corneal infections, especially to prompt diagnosis and treatment and prevent complications such as corneal scarring and potential blindness [22]. Specifically, conjunctival imprints can reveal characteristic basophilic inclusions within epithelial cells, which are suggestive of elementary bodies, the infectious form of chlamydia [30]. Some cellular indicators of inflammation, specifically leucocytes, mast cells, and eosinophils, can be clearly detected under pathological conditions (PAS staining) and therefore not investigated for our healthy-control study population.

In comparison with the gold standards, the value of this new device, and particularly the accuracy produced in the sampling, provided a favorable response for its use for clinical practice outside the ophthalmological units and in the case of non-expert specialists.

Although the imprinting is now a current routine in several ophthalmological units, it is not so easy to obtain a good-quality sample. To bypass these problems, the group of Baudouin produced a CE-certified medical device, the EYEPRIM (an American patent application US2011/319789), able to perform an easy collection of conjunctival cells at the ocular surface [31].

First, the SurfAL device proved to be a handy, ergonomic, and extremely useful tool for ophthalmologists, particularly in terms of sampling capabilities and handling during the urgency of a clinical ophthalmic visit. In comparison to conventional devices, the results obtained first on sclerocorneal specimens and subsequently in a cohort of volunteers confirm the device’s easy-to-use properties. The possibility of obtaining appropriate and high-quality samples (cell populations with well-defined cellular margins) was the result of both handling and membrane properties (clear resolution under the optical field) that do not exhibit opacity and demonstrate statistically significant reproducibility [32]. Although slightly bulky for the patient and presenting a few additional difficulties, it significantly reduced the possible tremors of the operator’s hand, providing more stable samples for the final diagnosis [32]. By using the Acidic Phosphatases, PAS, muc5AC, and total proteins/RNAs evaluation, we observed that the SurfAL pen and the periocular-assisted SurfAL device provided imprints similar to those obtained with Millicell. It is noteworthy to stress that while PAS and muc5AC were tested as clear indicators of diagnosis/prognosis/monitoring, AP and total RNA/protein analysis had the purpose to validate the sampling procedure, providing information on the cellular processes of sampled epithelia, as measurement of lysosomal enzyme activity inside the cornea/conjunctiva; in fact AP is not an indicator of pathological state.

Since the main goal of those devices is the possibility to provide a quick response for driving practitioners or specialists to a good and suitable diagnosis and therapy, we compared the data obtained from the analysis of some indicators of immune cell recruitment/activation, inflammation, and ocular surface/tear film stability. These molecules, merely HLA-DR, ICAM-1, and p65-NFkB, were selected as working in concert to trigger cytokines that can, in turn, modulate the local inflammatory response. Data from this biomolecular analysis displayed that all devices have similar abilities to detect HLA-DR, ICAM-1, p65-NFkB, IL6, and muc5AC, which are all involved in cellular processes, particularly inflammation and immune responses at the ocular surface. These data reinforce the usefulness of SurfAL.

Applicability in clinical practice will include tear film changes associated with epithelial suffering and corneal ulcers, dry eye disease and squamous metaplasia, blepharitis, tear instability, OCP screening, and dry eye syndrome (Sjögren’s and non-Sjögren’s syndrome) [33,34,35,36].

The possibility of performing both microscopy and biochemical analysis, in addition to the molecular one, would allow identifying those candidate proteins and finding out differences in subtype distribution on the ocular surface. The ability to select different combinations of biomarkers will allow the device to be used in other strategic areas of great health, environmental, and commercial interest (the veterinary field, as well as cosmetic and agrifood industries). At the same time, a quick and safe outpatient screening would allow information about the pharmacological response and the efficacy of therapeutic application, an essential requirement in the ophthalmological practice. The flexibility of device allows the quick transfer of the impression cytology to the ophthalmological sanitary service and not only. Due to its easy handling, it might be useful also for forensic medicine and pre-banking screening of uninfected cornea or even for cyto-diagnosis of conjunctival epithelial monolayers for dry eye screening, differential diagnosis in case of suspected conjunctival infection, including the SARS-CoV2 ssRNA sequence or even in case of intact conjunctiva for investigating the presence of specific receptors, such as ACE2 receptor [37,38,39]. The all-in-one RNA/DNA/protein extraction from a single membrane might strongly depend on the type of sampling and the amount of cells, although it provides enough RNA (200–800 ng total RNA) and native proteins (1500 µg total proteins). Regarding morphological analysis, high-quality basal histology and immunofluorescence can be obtained, and the identification of cell subtypes as well as cellular position and source of biomarkers (ligand and receptors) in epithelial and goblet cells, and even inflammatory ones, can be carried out only by reducing the autofluorescence (quenching). PAS staining may represent a gold standard for this device, which is used as a point-of-care device. While the cytological staining can be carried out on-site with basal stainers, some doubts are still available for immunofluorescence due to the necessity of a UV lamp [37,38]. Also, the goal of carrying on the onsite molecular analysis (real-time PCR) is still not reliable for problems related to the necessity of real-time PCR.

The main limitation of this study is related to the small study population, which was related to volunteers adhering to the project aimed at evaluating the feasibility of this sampling with respect to morphological, biochemical, and molecular analyses.

The advantages related to this device are several and particularly related to the easy-to-use, speed in biosampling, non-invasiveness, and the miniaturization of the device that can also be used with refill and sample conservation.

Finally, the simple and elementary parts of SurfAL characterize the achievement of this device, and the gold standard method of sampling the ocular surface is guaranteed and improved by reducing time and patient discomfort. The standard method takes no more than 1 min to obtain an ocular imprint, and only 30 min is required for a simple colorimetric staining.

## 5. Conclusions

Traditional biosensors utilize biorecognition elements such as antibodies, enzymes, cells, and aptamers (amongst others) to capture and measure the targeted analyte. Empowering ophthalmologists with sampling devices that are easy and highly applicable in clinical practice will benefit any application in precision medicine, particularly those involving coupled digital analysis. The herein presented device is an open platform for analysis at any level, even outside the research laboratory (outpatient clinic, private or public study, consultants, hospital, and non-sanitary centers. The epithelial cell imprints provided by SurfAL, either alone or periocular-assisted, can safely be used as an implemented sampling procedure to ensure satisfactory results.

Taken together, the device has shown an effective, rapid, and non-invasive sampling and analysis of the ocular surface. Due to its size and shape, it was positively accepted by this small community of specialists (users) during their clinical routine and by volunteer subjects. However, the limit remains on the number of specialists interviewed. For this reason, further validation should be carried out in multicenter studies to establish its usefulness and promote its practice.

## 6. Patents

The device is a PCT (WO2016IB51474 20160316) and recently received the US 2018/0070929 A1 15 March 2018, “Device for sampling of the eye surface by imprinting” Inventors: Micera A, Zollo L, Ghezzi I, Balzamino BO, Sgrulletta R. Applicants: IRCCS-Fondazione Bietti and Campus BioMedico, Rome, Italy.

## Figures and Tables

**Table 1:** List of antibodies and primers.

**A:** Western and Immunofluorescence				
Target: protein	dilution	Host	Specificity	Source
Ck12	1:100	mouse	Corneal/limbal marker	Dako
Ck19	1:100	mouse	Conjunctival marker	Dako
Muc5AC	1:200	mouse	Goblet Cell Mucin	Santa Cruz
AP	1:1000	rabbit	Ocular surface indicator	Abcam
Actin	1:1000	mouse	Cytoskeleton marker	Abcam
**B**: molecular analysis				
Target: transcript	Accession	Sequence (left primer)	Tm/amplicon	
Hu Muc5AC	AF015521	tcc acc ata tac cgc cac aga	59 °C/103 bps	
Hu IL6	BC015511	gac agc cac tca cct ctt ca	60 °C/125 bps	
Hu ICAM-1	J03132	atg agt gcc cag gga ata tg	59 °C/107 bps	
Hu HLADR	V01511	ctg gcc aca ctg agg tgc at	60 °C/120 bps	
Hu p65-NFkB	L19067.1	cag aag cag gct gga ggt aa	60 °C/117 bps	
Hu GAPDH	BC013310	gaa ggg gtc att gat ggc aac	60 °C/111 bps	

Amplification profile: hot start activation (95 °C/15 min); 39 cycles: den. at 94 °C/10 s—ann. At 58 °C/15 s—ext. at 72 °C/10 s; melting curve recording 55 °C to 95 °C with one fluorescence reading every 0.5 °C; further ext. 75 °C/5 min; Legend: CK12/19, cytokeratins 12/19; AP, Alkaline Phosphatase; IL6, interleukin 6; ICAM-1, intercellular adhesion molecule-1; HLADR, Human Leukocyte Antigen Determinant R; p65-NFkB, Nuclear Factor kappa-light-chain-enhancer of activated B cells; GAPDH, glyceraldehyde-3-phosphate dehydrogenase.

## Data Availability

The original contributions presented in this study are included in the article. Further inquiries can be directed to the corresponding author.
